# A Review of Genetic and Gene Therapy for Parkinson’s Disease

**DOI:** 10.7759/cureus.34657

**Published:** 2023-02-05

**Authors:** Omkar Dumbhare, Sagar S Gaurkar

**Affiliations:** 1 Medicine, Jawaharlal Nehru Medical College, Datta Meghe Institute of Higher Education and Research, Wardha, IND; 2 Otolaryngology - Head and Neck Surgery and Surgical Oncology, Jawaharlal Nehru Medical College, Datta Meghe Institute of Higher Education and Research, Wardha, IND

**Keywords:** motor symptoms, gene therapy, levodopa, snca protein, parkinson’s disease

## Abstract

Parkinson’s disease (PD) is a syndrome with deterioration of neurons, with its onset starting in the ’20s, known as the young beginning of Parkinson’s to the late inception of the ailment in the 60s. The majority of the environmental risk associated with PD is age. The pathophysiology of PD is related to the accretion of synuclein alpha (SNCA) protein leading to toxicity. This toxicity further leads to a depletion in dopamine levels, creating both motor and non-motor symptoms. PD is the combination of genetic and environmental risk factors. Linkage and association studies provided data on autosomal dominant and recessive genes linked to PD. Current treatment regimes involve using levodopa, catechol-O-methyl transferase inhibitors, anticholinergics, and monoamine oxidase B (MAO-B) inhibitors. Genetic treatment is done by identifying possible targets. Gene therapy includes silencing, replacing, or correcting the flawed gene with a good gene. This therapy has the advantage of eliminating significant PD symptoms with fewer to no adverse effects than conventional treatment. These targets are organized into disease-modifying or non-disease modifying. The distinction between these two is that disease-modifying treatment stops the degeneration of neurons, while non-disease modifying treatment involves dopaminergic enzyme expression. In non-modifying targets, aromatic L-amino acid decarboxylase (AADC) therapy is used but not as a standalone, so the presentation of AADC, tyrosine hydroxylase (TH), and GTP cyclohydrolase 1 (GCH) is done together as a tricistronic system. With these developments, a drug named prosavin is under clinical phase 1 trial. Disease-modifying targets involve glial cell-derived neurotrophic factor (GDNF). Direct GDNF delivery reduces PD symptoms. This GDNF infusion technique works with a tetracycline-controlled transactivator. Gene therapy introduction into the treatment of PD would be beneficial as there would be lesser adverse effects seen as linked with conventional treatment involving levodopa, MAO-B inhibitors, and anticholinergics, among a few. This article discusses the genetic basis and genetic model of therapy for PD.

## Introduction and background

Following Alzheimer’s, Parkinson’s disease (PD) has the highest number of neuronal degeneration among all neurodegenerative disorders. It is a long-lasting, advanced neurodegenerative ailment described by the premature death of dopaminergic neurons in the SNpc and the widespread occurrence of alpha-synuclein, an intracellular protein. Lack of dopamine in the basal ganglia advances motor symptoms [1]. The association of PD with non-motor symptoms precedes motor indications by more than ten years. Non-motor symptoms are more challenging to manage in the future phases of PD. Development of disabling features includes non-motor signs, dopamine-resilient motor symptoms, and motor complications due to long-standing dopamine therapy. Other than the fundamental symptoms (akinesia and bradycardia, rigidity, and tremor), Parkinson's patients display supplementary motor difficulties: gait trouble, speech arrears, grip force, and compromised handwriting, among others. Several problems respond to treatment with dopaminergic medications and deep brain stimulus. Non-motor signs associated with Parkinson's include complaints of loss of interest, depression and anhedonia, hallucinosis, and cognitive impairment, among a few. All these symptoms are tagged along with disturbances in the sleep-wake cycle regulation [2].

The pathophysiology of PD includes the cellular accretion of SNCA in insoluble particles that cause cellular impairment and toxicity. The assembly of SNCA is evident with the depletion of dopaminergic neurons, which disrupts numerous pathways depending on the dopaminergic tonus in the Substania Nigra pars compacta. This imbalance causes striatal projections to deviate in the neuronal circuits contributing to the parkinsonian phenotype. The accretion of SNCA is also a pathologic symbol depicted in numerous brain diseases. Among the few are Lewy body dementia and multiple system atrophy [3]. PD, predominantly, is caused by an amalgamation of genetic and environmental risk components. PD is assumed to be multifactorial rather than the pathogenesis of a single definite cause. Aging is the most significant environmental risk component [4]. Neurodegeneration is closely associated with aging, which leads to the progression of PD. Selective damage to dopaminergic neurons has been done by toxins. Limited protective components such as nicotine, caffeine, and non-steroidal anti-inflammatory drugs (NSAID) have also been acknowledged in the classical epidemiological study of early lifestyle changes prolonging Parkinson's [5].

## Review

Methods

This systematic review adhered strictly to the guidelines outlined in the Preferred Reporting Items for Systematic Reviews and Meta-Analyses (PRISMA). Data collection was conducted in September 2022 by searching multiple electronic databases such as PubMed, Google Scholar, ScienceDirect, PubMed Central, Cochrane Data, and the website of Neuropathology using keywords such as "Parkinson's disease," "cognitive impairment," "genetics of Parkinson's," "gene markers," and "Parkinson's genetic therapy," both individually and in combination. A total of 2,191 articles were identified using these keywords. The articles were screened by reviewing their titles, abstracts, and relevance to the research question. Inclusion and exclusion criteria were applied to further narrow down the articles to those that were relevant to the study. A quality appraisal was performed for all the articles using established guidelines, and 42 high-quality articles were included in the final analysis. The databases were last accessed on September 2022.

Search Sources/Search Strategy

In PubMed, Google Scholar, ScienceDirect, PubMed Central, Cochrane data, and the website of Neuropathology, we used the MeSH strategy we obtained: ("Parkison's/disease" [Majr] OR "Parkinson's/features" [Majr] OR "Parkinson's/genes" [Majr] OR "Parkinson's disease/genetics" [Majr] OR "Parkinson's disease/mutations" [Majr] OR "Parkinson's disease/autosomal dominant" [Majr] OR "Parkinson's disease/autosomal recessive" [Majr]); for the treatment it was: ("Treatment/drugs" [Majr] OR "Genetic diagnosis/therapy" [Majr] OR "Parkinson's disease/gene therapy" [Majr] OR "Genetic therapy/AADC" [Majr] OR "Genetic therapy/GDFN" [Majr] OR "Gene therapy/disease modifying" [Majr] OR "Parkinson's therapy/non-disease modifying" [Majr]). 

We obtained the most pertinent research papers, and we used them in different arrangements with the Booleans ''AND,'' ''OR.'

Inclusion Criteria 

The study selection criteria included peer-reviewed articles published in the last 35 years, in English, that were relevant to our topic and research question and involved human participants over the age of 50. The types of studies included were clinical trials, observational studies such as case-control, cohort, and cross-sectional, as well as systematic reviews, meta-analyses, and literature reviews.

Exclusion Criteria

The study selection criteria also included non-peer-reviewed literature such as grey literature, books, letters to the editor, and editorials. However, studies that were deemed to be duplicates or overlapping were excluded, as well as in vitro studies or studies that were conducted on animals.

Result

**Figure 1:**
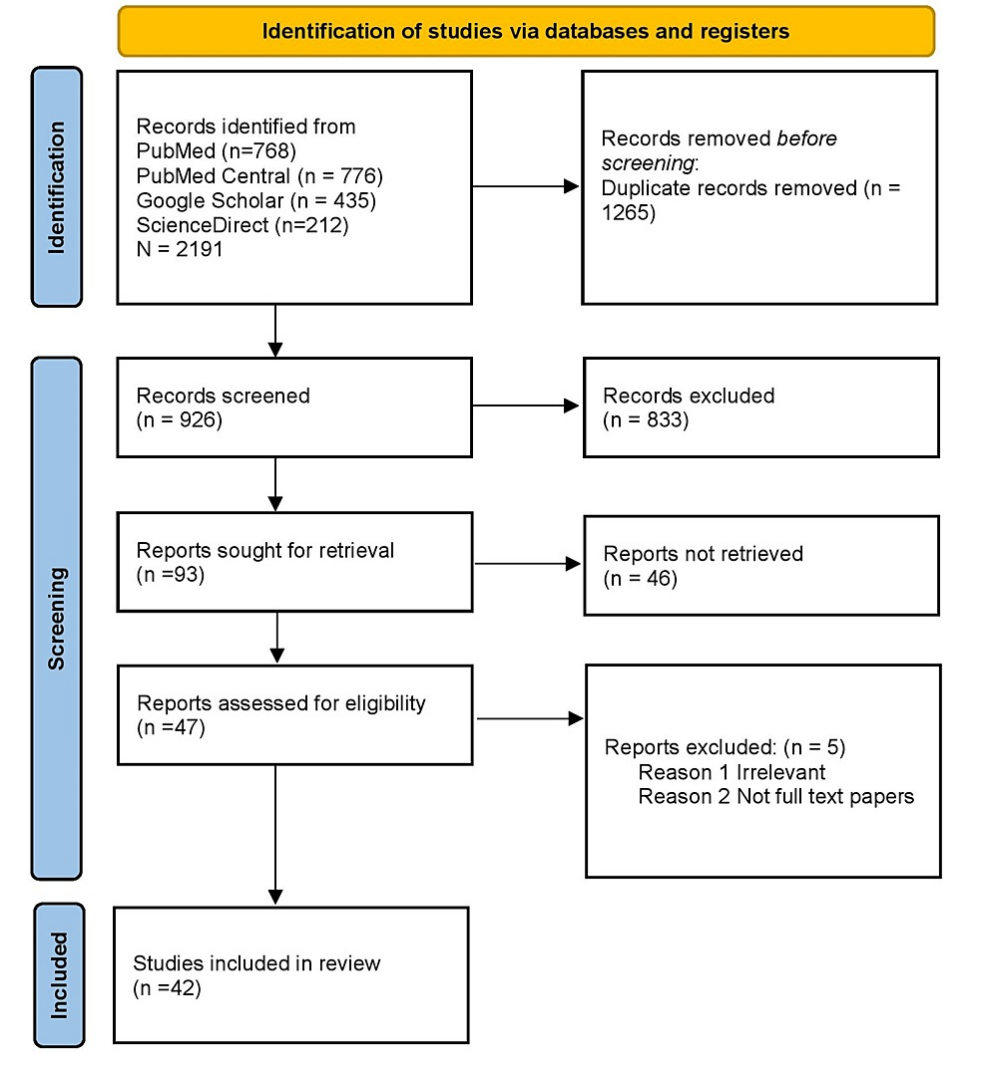
PRIMSA 2020 flow diagram for the systematic review PRIMSA: Preferred Reporting Items for Systematic and Meta-analysis, PMC: PubMed Central.

A total of 2,191 studies were initially identified from databases, but after applying filters based on inclusion criteria such as being in English, articles from the last 35 years, and involving humans, clinical trials, all types of reviews, and observational studies, the number of studies was reduced to 926. After further screening and quality appraisal, the number of studies included in the study was reduced to 42, including one article from the website of neuropathology. The study found that 30 studies provided evidence of a relationship between genetics, gene mutations, age, and Parkinson's disease, with 12 studies specifically finding that genetic mutations in SNCA, LRRK2, PARK2, PINK1, DJ1, GBA, and MAPT alleles are associated with the disease (Figure 1).

Genetics of PD 

Findings from numerous disease-instigating genes and genetic risk components have validated genetic involvement in studying the pathophysiology of PD [6]. Linkage studies help identify genes triggering monogenic varieties of ailments and analyze families with several relatives expressing a rare pathogenic mutation in the gene isolating the disease [7]. Association studies identify genes linked with risk factors. The latter is much more common when comparing the data between high-trenchant disease-causing transmutation and genetic risk variants. Association studies prerequisites inclusion of large cohorts of patients and control, as genetic risk variants have a low effect strength. Genome-wide association studies provide us with an unbiased approach [8]. Data obtained from linkage analyses and association studies have identified numerous loci (EIF4G1, PARK13, PARK15, and PARK1) and risk components (HLA, GAK, MAPT, BST1, PARK16, GBA, and LRRK2, SNCA), respectively [9]. A significant amount of data has shown the risk for PD originated from genetics. These disease-causing genes are of various classes [6]. Sporadic PD closely resembles the phenotype of the dominant form of genes. At the same time, recessive variants (ATP13A2, PLA2G6, and FBXO7) show earlier onset age and represent pure Parkinsonism along with additional clinical features. More than 20 genes have been identified causing autosomal recessive (AR), autosomal dominant (AD), and X-linked parkinsonism, along with specific phenotypes varying from idiopathic PD-like to early commencement of Parkinsonism, which can be pure or can be intricated by motor and non-motor clinical characters [10].

Autosomal dominant PD genes 

The initially identified PD-causing gene was SNCA (PARK1/PARK4). Mutation of SNCA is responsible for autosomal dominant PD. The central unit of Lewy bodies and Lewy neurites is protein alpha-synuclein coded by the gene SNCA. This protein alpha-synuclein possibly helps with brain plasticity and binding to synaptic vesicles. Expression of this protein is widespread in the brain localizing to presynaptic nerve terminals. Naturally, these proteins are present unfolded but can oligomerize, resulting in the formation of fibrillar components, which are the elements of Lewy bodies [11]. In high concentrations, the alpha-synuclein protein of wild type appears to be lethal to neurons. According to the gain-to-function hypothesis, a point mutation in SNCA leading to translational changes in alpha-synuclein protein might increase protein aggregation. Patients with SNCA duplications feature late-onset PD phenotypes with sluggish development and no uncharacteristic features. Patients with an SNCA triplication or the A53T missense transmutation have a prior age at commencement (around 34 years), and transporters of a triplication, the E46K or A53T missense mutation, existing commonly with dementia than duplication carriers or carriers of the A30P missense transmutation. This gene was acknowledged as unsafe in all genome-inclusive association studies. The variants allied with the amplified hazard of PD might hint to some extent at a higher alpha-synuclein manifestation [12].

Transmutation in another gene known as the LRRK2 gene also causes autosomal dominant PD. Protein translated by LRRK2 is suggestive of a function in intracellular signaling pathways. Expression of this gene is widespread in the brain localizing to vesicles and membranes (lysosomes, endosomes, and mitochondria). Mutation in LRRK2 leads to PD due to increased kinase activity, degrading changes in the ability to dimerize, and impairment in the GTPase functionality. The Prime sign in LRRK2 mutated PD is tremor. Patients with LRRK2 give the impression of more benign growth of the disease with sluggish development but a minor prevalence of dementia and psychiatric difficulties. LRRK2 mutation carriers pathologically show signs of alpha-synuclein Lewy bodies. In contrast, a small fraction shows anomalous pathology such as tau pathology, neuronal loss deprived of intracellular inclusions, diffuse Lewy body illness, and motor neuron disease. The exact pathogenic mutations with diverse pathological variations can be seen midst members of the same family [13].

Autosomal recessive PD genes 

Autosomal recessive PD is triggered by transmutation in the PARK2 (Parkin) gene. The protein Parkin belongs to ubiquitin E3 ligases. This protein assists in the role of mitochondrial maintenance and can cause autophagy of non-functioning mitochondria. Parkin protein catalyzes the bonds between ubiquitin and proteins by associating with ubiquitin-conjugating enzymes (E2s). Expression of this protein can be seen in many tissues, which include the brain and substantia nigra, localizing to the cytosol, Golgi complex, ER, and the outer mitochondrial membrane. Patients with Parkin mutations tend to develop motor variations and dyskinesias in the early itinerary of therapy for levodopa-responsive PD. Patients also show dystonia in the lower extremities along with psychiatric abnormalities at the onset of the disease [14].

A mutation in the PINK1 (PARK6) gene also instigates autosomal recessive PD. The protein encoded assists in cellular and oxidative stress function with the help of the mitochondrial response. The protein encoded by the translation of this gene is also located in the mitochondrial membranes of the brain. PINK1 is assumed to shield neurons from proteasome-stimulated apoptosis and mitochondrial malfunction by interacting with Parkin. The mutation in this gene impairs the protein's function, leading to rare cases of Parkinson’s with an early onset of dementia. At the same time, some patients have also shown additional psychiatric disturbances, including anxiety and depression [15].

A mutation in DJ1 (PARK7) is yet rare and another cause for the causation of autosomal recessive PD. The protein DJ1 seems neuroprotective and functions as a sensor for oxidative stress. This protein also acts as an antioxidant under the action of the mitochondrial membrane during oxidative stress [16]. This protein activity is significant in neurons of the nigra strata as they are more vulnerable to oxidative stress, which explains the mutation in genes leading to PD. Expression of DJ1 is widespread in various tissues, as well as the brain, neuronal, and glial cells [17]. Parkinson's caused due to mutation in DJ1 accounts for rare early-onset cases, as clinical representation shows a helpful reaction to levodopa and sluggish progression of the disease [18].

Genetic risk factors

Several risk factors are known to exist. One of them is Gaucher disease, triggered by homozygous or compound heterozygous mutations in the glucocerebrosidase (GBA) gene. In relation to this data, single heterozygous mutations are significant risk factors for PD. Gaucher disease is an autosomal recessive inborn error of metabolism, a rare lipid storage disorder manifesting complications including the nervous system, liver, bone marrow, spleen, and lungs [19]. Patients with Gaucher disease seem to advance to PD [20]. Gaucher is a lipid storage disorder where glycolipids and glucosylceramide accumulate in the lysosomal component. Families of Gaucher disease patients have greater chances of advancing to PD. Patients with the GBA mutation show symptoms of severe nonmotor and cognitive variations. Bradykinesia is the initial symptom, along with levodopa dyskinesia [21-23].

Frontotemporal dementia is caused due to mutations in the microtubule-associated protein tau (MAPT), an identified risk component for advancing supranuclear palsy and corticobasal deterioration. MAPT gene encodes for protein tau. The role of tau is to maintain the integrity of the cytoskeleton. PD is due to the accretion of alpha-synuclein. A suggestion of interaction linking these two pathophysiologic mechanisms of PD and Tau inclusions in various neurodegenerative diseases has been linked [12].

The sporadic late onset of PD with BST1 as a risk element in the Asian genome-inclusive association study showed that allele frequencies varied among studies, where effect sizes seemed lower in the European-origin population. A protein expressed from this gene functions as a catalyst for the growth of cyclic ADP-ribose and the maintenance of Ca2+ homeostasis. PARK16 contains five genes with a locus on chromosome 1q32, representing an association with PD [24].

**Figure 2:**
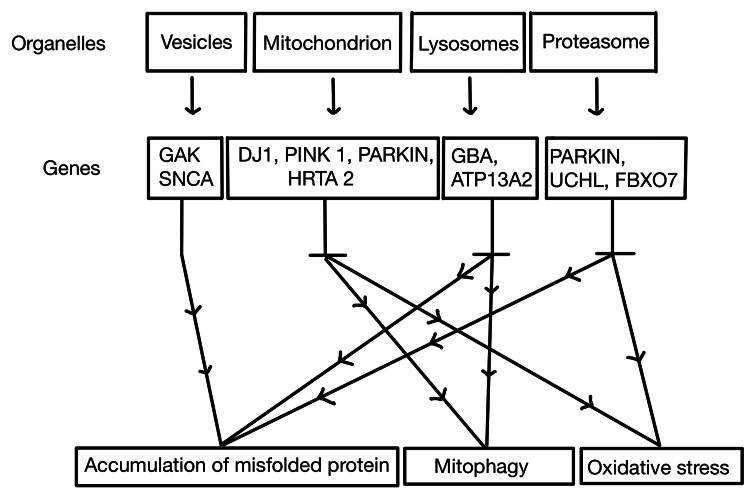
Pathophysiology of PD with the expression of proteins from various genes.

Figure 2 shows both autosomal dominant and recessive genes involved in the etiopathogenesis of PD where organelles such as vesicles, mitochondrion, proteasome, and lysosomes with the genes GAL, SNCA, DJ1, PARKIN, GBA, HRTA 2, and FBXO7 on mutation form accumulations of misfolded proteins.

Treatment and management of PD

Present-day PD treatment regimens, to name a few, include levodopa administration, dopamine agonists, deep brain stimulation (DBS) surgery, neuroablative surgery, and monoamine oxidase B (MAO-B) inhibitors. Current treatments are symptomatic and associated with side effects. Treatments provided do not stop the development of the disease, leading to more difficulties with increasing age. Aging is the most frequent risk factor among environmental factors for developing PD. Even though the current treatments are symptomatic and associated with side effects, they offer substantial symptomatic alleviation of the significant motor symptoms. For non-motor manifestations, these non-disease-modifying drugs offer little clinical relief. Underdiagnosis of Parkinson's, the treatment is delayed until the symptoms become problematic for the patient to diminish the effect of the drug's antagonistic effects [25].

The backbone of existing Parkinson's treatment is levodopa-based medications. The mechanism followed by levodopa-based preparations replaces the dopamine depleted in the striatum. Dopamine cannot cross the BBB, while its precursor, levodopa, can cross the BBB and is therefore used in therapy. After the levodopa crosses the BBB, it is converted into the neurotransmitter dopamine by DOPA decarboxylase [26,27]. At first, a low dose of levodopa is administered, which is further tittered up depending on dosage side effects. Patients are administered multiple doses of levodopa, ranging from 150 to 1000 mg daily. Increasing doses also increase the adverse effects associated with levodopa [28]. Adverse effects of levodopa consist of peripheral external to CNS conversion by DOPA decarboxylase leading to significant motor complications [27]. These peripheral, outside-the-CNS conversion complications are counteracted by dispensing levodopa in a mixture with peripheral inhibitors of DOPA carboxylase. Dopamine agonists are the most commonly used drugs. One of them is the agonist ethanolamine, classified into ergot and non-ergot derivatives. The mechanism of these agonists is by attaching to the dopaminergic receptors. Particularly in younger patients, dopamine agonists have initially been prescribed as medications for therapy. The advantage of this approach is the delay in using levodopa, which reduces the antagonistic effects of motor complications. Some drugs were withdrawn from the therapy due to significant idiosyncratic antagonistic effects. For example, pergolide use presented a risk of retroperitoneal, pericardial, and pleural fibrosis. This drug was withdrawn from treatment in 2007 [29].

MAO-B inhibitors are special chief enzymes implicated in the itemization of dopamine. These drugs follow inverse relations with dopaminergic activities within the striatum. As the MAO-B inhibiting enzyme decreases, dopaminergic activity increases. Sometimes this drug class is sufficient to relieve the early motor symptoms of PD. These drugs, combined with levodopa, are used to lower levodopa dosage concentration. Gastrointestinal problems are most familiar with MAO-B inhibitors [30]. Anticholinergic drugs are also administered in PD. These act through non-dopaminergic mechanisms, while drugs like levodopa preparations and MAO-B inhibitors increase dopaminergic activity in the striatum. Anticholinergics work by reducing the neurotransmitter acetylcholine activity. These drugs' primary function is to relieve mild motor symptoms, notably tremors, and muscle stiffness. Anticholinergics are used in combination with levodopa. These drugs are avoided in elderly patients with cognitive complications [31-33].

Amantadine is another drug used in PD. The development of amantadine was done mainly for treating flu, but this antiviral drug has been used to treat various indications of PD. Symptoms especially involve rigidity and rest tremors. Like levodopa, amantadine is started at a lower dose and titrated up. Side effects of this drug include insomnia, nightmares, sweating, and headaches [34].

Gene therapy for PD 

In basic terms, gene therapy means "replacing bad DNA with good DNA" [25]. The sophisticated definition of gene therapy can be described as the overview of beneficial genes substituting, muzzling, or amending faulty genes. This engineering mechanism involves using non-replicating viral vectors and numerous recombinant adeno-associated virus (AAV) serotypes [33]. Murine PD models have shown successful utilization of gene delivery [34,35]. The genetic treatment of PD involves treating known possible targets. These targets can be categorized into disease-modifying or non-disease-modifying. Non-disease-modifying treatment can be done by normalizing anomalous firing in the basal ganglia with the expression of dopaminergic enzymes. This particular set of treatments is symptomatic and does not involve any alteration in the pathophysiological process. Disease-modifying treatment can be done by stopping Parkinson's mediated cell and regeneration of neurons. Neuroprotective properties can be found in the overexpression of growth factors [36].

Non-disease modifying targets

The transformation of L-DOPA into dopamine is done by a synthetic dopamine apparatus with the help of the enzyme aromatic acid decarboxylase (AADC). AADC therapy is not suitable as a standalone treatment as there is a constant requirement for exogenous L-DOPA. During the trials, the patients have reported better L-DOPA responses [37]. Another method to review symptomatic PD is entirely reconstructing the dopamine synthesizing apparatus by instituting certain genes. These genes will assist the three target genes' enzymes in converting tyrosine to dopamine, removing the need for exogenous L-DOPA and ensuring stable dopamine tonus. Including TH, AADC, and GTP cyclohydrolase 1 (GCH) would eliminate off-target effects and wearing-off effects [37]. Delivery of these three enzymes is an issue because, when they are combined, the giant molecule does not fit into a single AAV genome. The solution is inoculation by combining three different AAV vectors. The use of tricistronic vectors gave results of overexpression of GCH, TH, and AADC. To ensure there is no hassle with tricistronic system packaging, all the genes are done in one vector to ensure joint expression of the proteins. Herein the LV approach was efficient in rats. The therapy was well tolerated in monkeys with noticeable development of parkinsonian symptoms with no signs of dyskinesias. On microdialysis, 50% restoration was seen of normal dopamine levels. One such drug named Prosavin, with the preparation LV-TH-GCH-AADC, is under phase 1 clinical trial [38].

As the striatal dopaminergic tonus decreases, the excitatory activity of the circuits in the STN-glutamatergic subthalamic nucleus increases. Increased firing frequency affects the depolarization of STN along with the spike burst after each hyperpolarization. Inhibitory effects on the thalamocortical circuits due to amplified activation of GPi and SNr results in Parkinson’s motor symptoms. Studies have found that GAD overexpression in rats eliminated parkinsonian motor symptoms and was well tolerated. A company named Neurologix funded the phase 1 clinical trial that came with the results of improvement in UPDRS scores and no adverse effects in both ON- and OFF-state. As of the current date, the study and improvement of GAD as a conceivable gene therapy for Parkinson's has come to an end as the company named Neurologix has dissolved [39].

Disease-modifying targets

SN and striatum receiving direct glial cell-derived neurotrophic factor (GDNF) delivery diminish Parkinsonian symptoms in toxin models of Parkinsonism along with neurite sprouting. Insufficient spreading of the drug has been linked to a lack of GDNF infusion which may be on account of pump malfunction in addition to a deficient amount of neurons for GDNF to affect. Neurotensin-polyplex, a transfection method of GDNF in dopaminergic cells, is a viral vector delivery. This method works by presenting nanoparticles encompassing plasmids for the expression of GDNF with the help of neurotensin receptors in dopaminergic cells. The dopaminergic cells are under the control of a tetracyclin-controlled transactivator. The mechanism of this method functions with the supply of tetracycline, which enables the controlled expression of GDNF, which is otherwise controlled by the need for exogenous stimulation. Another method for this GDNF gene transfer was using nanoparticles made of 30-mer lysine polymer and a plasmid encoding GDNF. In this GDNF overexpression therapy conducted in MPTP-treated monkeys, a safety concern was raised when viral vector-mediated GDNF was inoculated in the SN with AAV-GDNF. There were no signs of motor development, but rather significant weight loss was observed as an adverse effect. FDA has permitted the American phase 1 trial on progressive Parkinson's patients [40].

## Conclusions

Following Alzheimer's, PD has the highest number of neuronal degeneration among all neurodegenerative disorders. Deficiency of dopamine in the basal ganglia precedents motor symptoms, viz, later postural unsteadiness, rigidity, tremor, and bradykinesia. PD in most cases is instigated by an amalgamation of genetic and environmental risk components. PD is assumed to be multifactorial rather than the pathogenesis of a single definite cause. Findings from numerous disease-causing genes and genetic risk factors have validated genetic involvement in studying the pathophysiology of PD. Data obtained from linkage analyses and association studies data analyses have shown the association of various loci (EIF4G1, PARK13, PARK15, and PARK1) and several risk factors (HLA, GAK, MAPT, BST1, PARK16, GBA, LRRK2, and SNCA), respectively. A mutation of SNCA is responsible for autosomal dominant PD. The principal unit of Lewy bodies and Lewy neurites is the protein alpha-synuclein, which is coded by the gene SNCA. This protein, alpha-synuclein, possibly helps with brain plasticity and binding to synaptic vesicles. Autosomal dominant PD is also triggered by a mutation in the LRRK2 gene. Proteins translated by LRRK2 are suggestive of a function in intracellular signaling pathways. Autosomal recessive PD is instigated by transmutation in the PARK2 (Parkin) gene. The protein Parkin belongs to ubiquitin E3 ligases. Patients with Parkin mutations tend to develop motor variations and dyskinesias in the initial sequence of treatment of levodopa-responsive PD. Autosomal recessive PD is also caused by a mutation in the PINK1 gene. The mutation in this gene impairs the function of the protein leading to the rare case of early onset of Parkinson's with early onset of dementia. Mutation in DJ1 is yet rare and another cause for the causation of autosomal recessive PD. Genetic risk factors in connection with Gaucher to PD with association GBA gene. Under treatment, the primary and major treatment is to be done with levodopa preparations. Levodopa preparations give adverse effects with increased titer doses. In combination with levodopa, MAO-B inhibitors and anticholinergics are also given depending on the symptoms of the disease. Gene therapy is replacing, silencing, or correcting bad DNA with good DNA. Gene therapy is classified under disease-modifying targets and non-disease-modifying targets. Disease-modifying targets such as treatment with GDNF genes and non-disease-modifying targets such as treatment with TH, AADC, and GCH genes among others.
